# Development of Growth Media from Agricultural By-Products for Cultivation of PUFA-Producing *Sicyoidochytrium minutum*

**DOI:** 10.3390/md20010008

**Published:** 2021-12-22

**Authors:** Heiðrún Eiríksdóttir, Magnús Örn Stefánsson, Hjörleifur Einarsson

**Affiliations:** 1 BioPol ehf, 545 Skagaströnd, Iceland; heidrun@ni.is (H.E.); mstefansson@fas.harvard.edu (M.Ö.S.); 2 Iceland Institute of Natural History, 210 Gardabaer, Iceland; 3 Department of Human Evolutionary Biology, Harvard University, Cambridge, MA 02115, USA; 4 Faculty of Natural Resource Sciences, School of Business and Science, University of Akureyri, 600 Akureyri, Iceland

**Keywords:** Thraustochytriaceae, DHA, EPA, peptone, single-cell oil

## Abstract

The demand for novel sources of marine oils, which contain polyunsaturated fatty acids (PUFAs), has increased due to the realization of the importance of PUFAs, e.g., docosahexaenoic acid (DHA), in the human diet. However, the natural supply is limited. By-product peptones (BYPP) intended as a growth medium for the PUFA-producing strain *Sicyoidochytrium minutum* of family Thraustochytriaceae were produced after several experiments on the pancreatic digestion of bovine lungs and spleens. *S. minutum* was able to grow in a medium containing BYPP made from the pancreatic digestion of lung and spleen with glycerol, resulting in 1.14 ± 0.03 g cell dry weight (CDW)/L and 1.44 ± 0.24 g CDW/L, respectively, after 5 days of incubation at 25 °C, compared to 1.92 ± 0.25 g CDW/L in Basal Medium (BM) containing tryptone, peptone, and glycerol. The lipid content, obtained after growth in lung BYPP media with glycerol as a carbon source, was significantly higher (28.17% ± 1.33 of dry weight) than in the control basal medium (BM) (21.72% ± 2.45); however, DHA as a percentage of total fatty acids was lower in BYPP than in the control BM (25.24% ± 1.56 and 33.02% ± 2.37, respectively). It is concluded that low-value by-products from abattoirs can be used as ingredients for the cultivation of oligogenic Thraustochytriaceae.

## 1. Introduction

Marine oils are important ingredients in food, feed, and as dietary supplements (e.g., omega-3 fatty acids). The natural supply of marine oils and omega-3-rich oils is from fatty fish. However, as this supply is limited and there is a growing demand for marine oils (e.g., for aquaculture feed) [1], novel sources are needed. Marine algae and heterotrophic protists have been identified as alternative candidates for the production of marine oils.

Both hetero- and phototrophic microorganisms produce lipids during their lifecycles, but the amounts and types of lipids vary between species. Phototrophic organisms, such as algae, use light as an energy source [2]. Light can be limited during winter at high latitudes, thus making heterotrophic microorganisms more attractive for biomass production. Thraustochytriaceae are heterotrophs that secrete extracellular hydrolytic enzymes, such as amylase, lipase, cellulase, and protease, and can utilize organic and decaying materials to produce oils [3]. Some strains can produce high amounts of long-chain polyunsaturated fatty acids (LC-PUFAs), e.g., omega-3 (*n* − 3) fatty acids such as docosahexaenoic acid (DHA) [2,4].

Thraustochytriaceae were first described by Sparrow [5], and exist in variable sea environments [2,4]. Thraustochytriaceae were originally classified within the kingdom Fungi, but genetically Thraustochytriaceae are not related to fungi, though they are chemoorganotrophic [6]. Thraustochytriaceae belong to the kingdom Chromista and class Labyrinthulomycetes. They are classified under phylum Stramenopiles (Heterokonts), which are characterized by zoospores with two differently sized flagella [2,3,7].

Different strains of Thraustochytriaceae have been used worldwide to enrich products with DHA, e.g., eggs, meat, and milk [8,9,10]. Either extracted oil or whole biomass from microorganism cultivation can be added to poultry feed and feed for dairy cows [10,11]. Previously, rotifers and Artemia nauplii, both important components of a live diet for fish in aquaculture, were fed with a spray-dried DHA-rich strain of *Schizochytrium* sp. [12]. The results showed that it was possible to enrich both rotifers and Artemia nauplii with DHA. The demand for PUFAs, e.g., DHA and arachidonic acid (AA), for the production of human diet products has increased due to greater knowledge of their essential roles in the functional development and growth of the brain [13].

The cultivation of heterotrophic organisms requires nutrients such as peptones, carbohydrates, vitamins, and minerals. These ingredients can be expensive and cheaper sources should be considered to lower the production cost [14]. By-products from food industries (agriculture and aquaculture) are rich in protein, carbohydrates, and fat, and could provide valuable ingredients for the cultivation of heterotrophic microorganisms. Several kinds of by-products have been used to cultivate oil-producing microorganisms, e.g., breadcrumbs [15,16], soymilk residues [17], organic waste from brewery operations [18,19], and sorghum juice [20]. By-products received from beef production are about 40% of live weight. Some parts of the by-products are disposed of, but other parts have been used as feed for fish farming and as mink feed [21]. By-products from abattoirs could similarly be used as a source of nutrients (e.g., peptones) for microbial growth media.

The aim of this project was to produce by-product peptones (BYPP) from abattoir material (lung, spleen, and pancreas), and to use the BYPP to cultivate the oil-producing strain *Sicyoidochytrium minutum*.

## 2. Results and Discussion

### 2.1. Preparation of BYPP and Protein Measurements for Growth Studies

**Figure 1:**
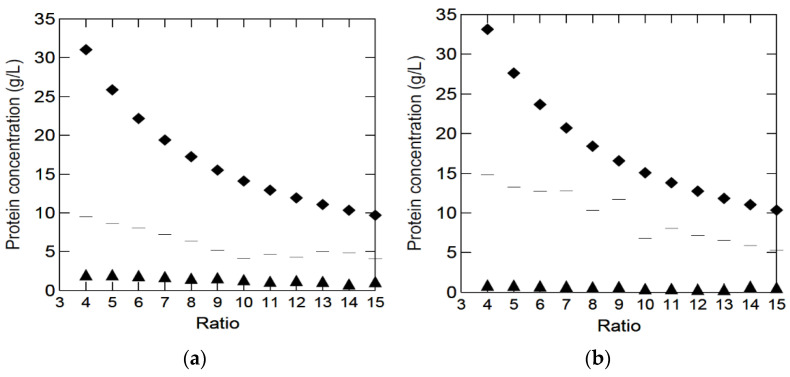
Peptide content in BYPP solutions obtained after pancreatic digestion of lung (**a**) and spleen (**b**) in lung:H2O ratios (1:4–1:15) estimated using OD_280_nm measurements (dashes ─) and Bradford‘s method (triangles ▲). Calculated maximum theoretical protein concentration (from Table 1) is also shown (rhombuses ♦).

After several experiments using different enzymes and technologies, a method for the production of a clear peptone solution for microbial growth media using the pancreatic digestion of bovine lungs and spleens was selected. The method was based on post-digestion filtration, heating, and centrifugation. Initial experiments showed better yields (g protein in solution/g raw material) in diluted solutions. More diluted solutions were easier to mix, thus giving the enzymes better access to the raw material. Therefore, pancreatic digestion was performed using different raw material/H2O ratios. The peptide contents in solutions with different by-products/H2O ratios are shown in Figure 1.

During the preparation of BYPP, especially through filtering using two-layered cheese cloth, undigested tissues and precipitation were filtered away, most likely containing protein, which explains why the maximum theoretical yield (g peptides in solution/g raw material) was not obtained. BYPP solutions were measured at an optical density (OD) of 280 nm, where three amino acids, cysteine, tyrosine and tryptophan, absorbs significantly at 280 nm [22]. Therefore, the Kjeldahl method, along with the OD_280_ nm measurements, were used to standardize the unknown BYPP solutions received after pancreatic digestion using linear regression, and this gave Equations (1) and (2). The difference between the protein content in the solutions and the initial protein content in the raw material decreased with increased dilution, indicating that the enzyme digestion was more effective in the more diluted solutions. For both lungs and spleen, the protein recovery was approximately 45% in the highest dilution (Figure 1).

Bradford’s method proved unsuitable for measuring the soluble protein content in these experiments (triangle in Figure 1). This may be because the BYPP solutions did not contain enough aromatic residues; therefore, the dye would not bind to the proteins, leading to an underestimation of the protein concentration [23]. It could also be that the pancreas was able to digest the by-products forming BYPP with proteins smaller than 3–5 kDalton; therefore, Bradford’s method would be unable to detect the proteins [24].

### 2.2. Growth Studies

**Figure 2:**
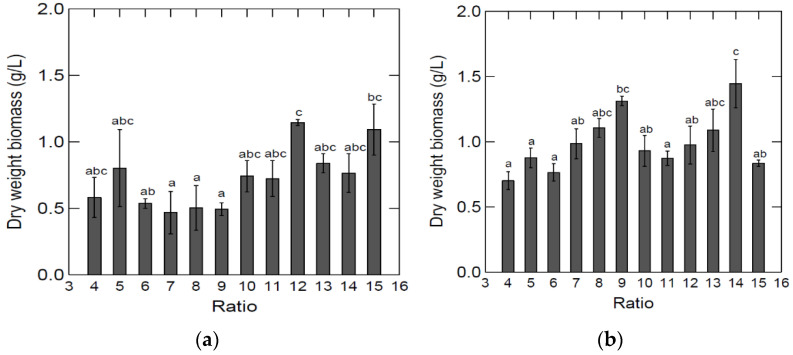
Average dry weight biomass using different lung (**a**) and spleen (**b**) to H2O ratios (grown at 25 °C for 7 days) in medium containing 2.18 (±0.25) g/L (lung BYPP) and 2.48 (±0.30) g/L (spleen BYPP). Sample size was *n* = 3 for each ratio. Standard deviation is shown in error bars. ANOVA test (*p* = 0.00194). The letters show statistically significant differences (*p* < 0.05, Tukey’s test) between measurements, where “a” shows the lowest value and “c” the highest.

The results from the growth studies can be seen in Figure 2.

Significant differences in biomass were obtained in media made from different ratios of by-products:H2O (Figure 2). Tukey’s test indicated that the highest dry weight biomass was in the ratio 1:12, and the four lowest dry weight biomasses were in the ratios 1:6, 1:7, 1:8, and 1:9. For growth in spleen:H2O media, the ratio 1:14 led to the highest biomass (Figure 2). It is not clear what the main source of variation between the dilution ratios was.

**Figure 3:**
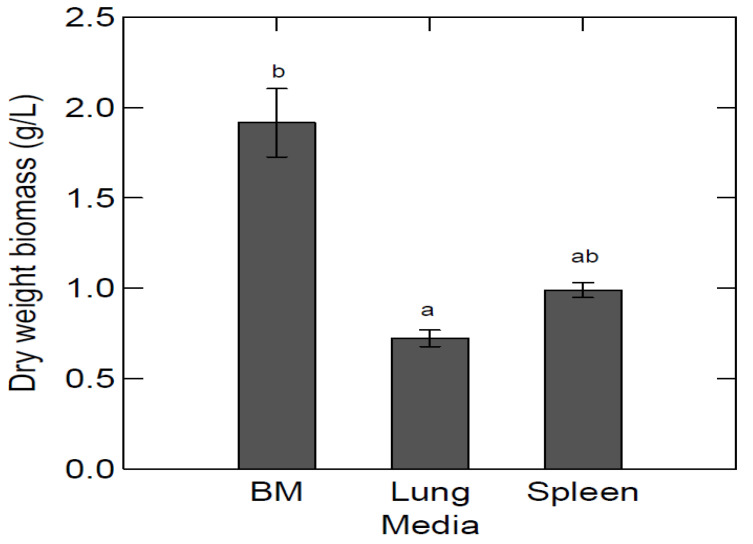
The average dry weight biomass in different media. The highest dry weight biomass in BYPP media was selected, lung:H2O ratio—1:12 and spleen:H2O ratio—1:14, and are compared to dry weight biomass in glycerol BM. Sample size is three for all media. Standard deviation is shown in error bars. ANOVA test (*p* = 0.00926). The letters show statistically significant differences (*p* < 0.05, Tukey’s test) between measurements, where “a” shows the lowest value and “b” the highest.

Figure 3 shows a comparison between the highest dry weight biomass obtained in BYPP media, with a lung:H2O ratio of 1:12 and spleen:H2O ratio of 1:14, and the dry weight biomass obtained in glycerol BM. These results indicate the utilization of abattoir by-products by *S. minutum*. The dry weight biomass was 1.44 (±0.24) g/L in media made with a spleen:H2O ratio of 1:14. No significant difference was observed between the spleen:H2O ratio and the BM (1.92 ± 0.25 g/L) dry weight biomasses. On the other hand, dry weight biomass in media made with a lung:H2O ratio of 1:12 was lower (1.14 ± 0.03 g/L) and significantly lower than the dry weight biomass in BM. There was no significant difference between the highest dry weight biomasses in media using the by-product:H2O ratio (Figure 3). Dry weight biomass after growth in spleen BYPP media was on average higher than the dry weight biomass obtained in lung BYPP media, indicating that the spleen was a better nitrogen source for *S. minutum* cultivation.

**Figure 4:**
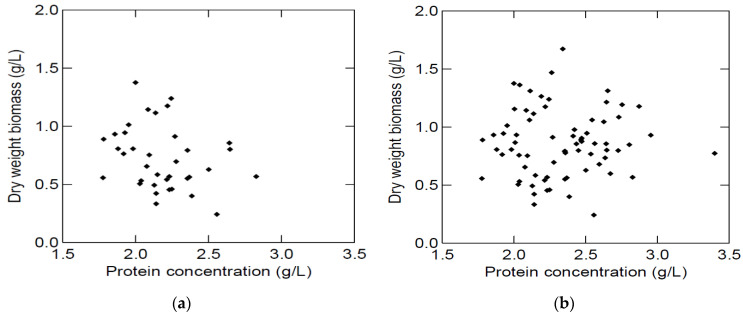
Effect of initial protein concentration on biomass production of *S. minutum* grown (5 d at 25 °C) in media made with lung BYPP (**a**) (*p* = 0.119. _adj_R^2^ = 0.043) and in media made with spleen BYPP (**b**) (*p* = 0.139. _adj_R^2^ = 0.036).

In spite of the attempt to have the same protein concentration at the start of each growth study, the initial protein concentrations varied and were on average 2.18 (±0.25) g/L in media made from lung BYPP and 2.48 (±0.30) g/L in media made from spleen BYPP. Therefore, the effect of the initial protein concentration on biomass production was tested (Figure 4). This initial variation did not significantly affect the final dry weight biomass. A possible explanation could be that these protein concentrations are close to a level that would be limiting to growth in the tested range (1.6–3.4 g protein/L) and growth conditions.

Several different types of by-products have been used for growth experiments in other studies. Some by-products contain nitrogen in the form of proteins, peptides, or free amino acids, whilst others include more carbohydrates. A by-product from the soymilk industry, called Okara powder, contained approximately 4.5% nitrogen and 47% carbon [17], which was calculated, with a Jonas factor of 5.71, to be equivalent to 25.7% protein [25]. Different strains of *Schizochytrium mangrovei* cultivated in media containing Okara powder for four days yielded a biomass of 7.51–7.86 g/L [16]. Thus, a greater dry weight biomass than that achieved in the current project was obtained, possibly because of the better growth conditions or due to different strains used. Okara powder media contained a protein concentration of around 2.5 g/L which was similar to the protein concentration used in this project (on average, 2.18 (±0.25) g/L for lung and 2.48 (±0.30) g/L for spleen). Soybean cake hydrolysate is also a cheap by-product which can be used as a nitrogen source. When used a substrate for *Schizochytrium limacinum*, dry weight biomasses ranging from 8.25 to 13.27 g/L were obtained, depending on the pH level and salinity[26].

### 2.3. Lipid Production

In growth media containing lung BYPP and glycerol, the test strain accumulated 28.2% (±1.33) lipids (dry weight) compared to 21.7% (±2.45) lipids (dry weight) in BM containing tryptone and glycerol. The by-products contained some fat but only minor amounts were found in the BYPP solution. These results are promising for the usage of by-products from abattoirs to produce lipids by *S. minutum* growth.

An important factor for growth and lipid accumulation is the C:N ratio. The N part is important for lipid-free biomass production and the C part later for lipid accumulation. In this study, the carbon part (glycerol) was 50 g/L, while the N part (tryptone, peptone or BYPP) was from 2.5 to 8 g/L. The lower peptone content, i.e., higher C:N ratio in the BYPP media could trigger lipid production. In addition, the higher lipid content in cells grown in BYPP media could be related to the slightly higher C content originating from the by-products.

**Figure 5:**
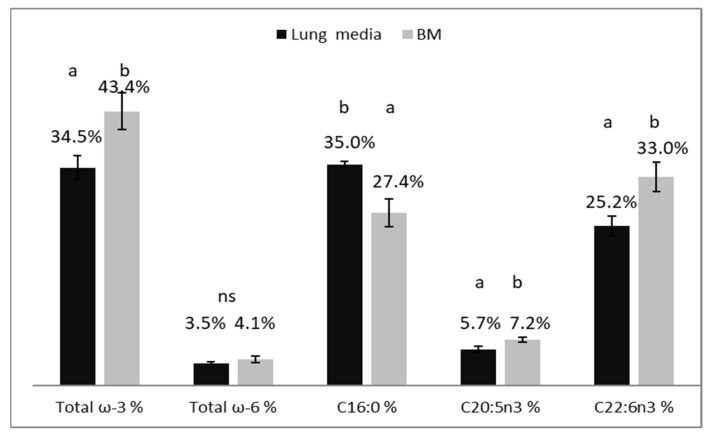
Percentage of ω-3, ω-6, C16:0 (palmitic acid), C20:5n3 (EPA) and C22:6n3 (DHA) fatty acids calculated from total fatty acid content (TFAC) in *S. minutum* strain grown in BYPP from lungs and in BM at 25 °C for 5 days. Dark column indicates results from lung media (*n* = 3) and light column from BM (*n* = 6). Standard deviation is shown in error bars. Different letters show statistically significant differences between columns (*p* < 0.05, Tukey’s test), where “ns” indicates no significant difference, where “a” shows the lowest value and “b” the highest.

The analysis of fatty acids showed that the *S. minutum* strain produced significant amounts of *n* − 3 fatty acids, both in BYPP media (34.48% ± 1.90 of total fatty acid content) and BM (43.43% ± 2.91). Most of the *n* − 3 fatty acid contents were DHA (C22:6n3): 25.24% ± 1.56 and 33.02% ± 2.37 in BYPP and BM, respectively. Eicosapentaenoic acid (EPA, C20:5n3) constituted 5.7% ±0.43 and 7.2% ± 0.33 in BYPP and BM, respectively (Figure 5). The test strain also accumulated considerable amounts of palmitic acid (C16:0): 34.96% ± 0.48 in BYPP medium and 27.35% ± 2.20 in BM. All the reviewed PUFAs were significantly higher in the BM, except for palmitic acid, which was higher in the BYPP medium. The pancreas digest contained very small amounts of C16 fatty acids, but it is unclear if this can fully explain the difference.

The lipid content and the amounts of PUFAs were higher in this experiment than in previous studies using the same test strain[27]. Other reports, using different strains and conditions, show that results on fatty acid production vary. Some [16] have reported higher amounts of DHA (31.1–41.1%) and palmitic acid (38.0–48.1%) in a *Schizochytrium mangrovei* strain grown in Okara powder residue, while others [19] found that a *Schizochytrium* sp. strain KH105 grown in barley shochu accumulated 25.8% DHA of total fatty acid content. The DHA yield after growth in soybean cake hydrolysate media was 18.45%, calculated from freeze dried cell biomass[26].

Peptones made from low-value abattoir by-products were shown to adequately support the growth of *S. minutum*, a *Thraustochytriaceae* strain. When included in a medium together with glycerol, the test strain produced significant amounts of PUFAs, especially DHA. Given the large strain variability of *Thraustochytriaceae*, other strains might show different results. Further studies are required to find out if it is economically feasible to produce peptone from animal by-products on a large scale for cultivation of Thraustochytrids. Additionally, further research should try to identify other sources of low-value ingredients (e.g., carbon-rich by-products) for microbial cultivation.

## 3. Material and Methods

### 3.1. Test Strain and Chemicals

The *S. minutum* test strain was obtained from BioPol Ltd.’s strain collection (ST30). For isolation and characterization of the strain, see [27]. The test strain was maintained and cultivated in a basal medium (BM) consisting of: glycerol or glucose (50 g/L); yeast extract (4 g/L); tryptone (4 g/L); KH2PO4 (2 g/L). Agar (15 g/L) was used to make solid medium when needed and tryptone was replaced with peptone when required. The ingredients were dissolved in 30% filtered natural sea water (Hunafloi, Iceland) and the solutions were adjusted to pH 7 (using HCl or NaOH) prior to sterilization (121 °C for 15 min). Penicillin and streptomycin (329.67 and 549.45 mg/L, respectively), were added after sterilization. Glycerol BM was also used in the growth studies as a control medium. All chemicals were purchased from Sigma-Aldrich (St. Louis, MO, USA).

### 3.2. By-Product Raw Material

By-products (intestines) were obtained from a slaughterhouse (SAH) in Blonduos (Iceland) and from a farm in Eyjafjardarsveit (Iceland). Lung and spleen were from cattle (*Bos taurus*) at SAH and pancreas was from sheep (*Ovis aries*) at the farm. Intestines, lung, spleen, and pancreas, were minced separately and stored at −18 °C. Pancreas was cooled in the mincing process to prevent autolysis.

### 3.3. Development of Digestion Method for By-Product Peptone (BYPP)

The by-products were homogenized, and the mince was dissolved in H2O and then treated in several ways (e.g., filtered, spun and incubated) in order to obtain a clear solution. Enzyme degradation was carried out using both commercial enzymes (Flavourzyme, Protamex, and Alcalase (Novozyme, Denmark)) and pancreas. The BYPP solutions were sterilized using an autoclave before the growth studies then centrifuged at 10,000× *g* for 5 min to remove precipitation. As pancreas showed satisfactory results in terms of protein yield and allowed the inclusion of more by-products, it was decided to only use pancreas for further studies, as described below.

### 3.4. Preparation of BYPP for Growth Studies

By-products (lung, spleen, and pancreas) were thawed at 4 °C overnight. Pancreas was used as an enzyme catalyst: four grams of lung or spleen was mixed thoroughly with four grams of pancreas and H2O in a glass beaker. Different ratios of raw material to H2O were used (1:4, 1:5, 1:6, 1:7, 1:8, 1:9, 1:10, 1:11, 1:12, 1:13, 1:14, 1:15). Three replicates were prepared for each digestion. The mixtures were incubated for 20 h at 50 °C with shaking at 150 rpm. The mixtures were then heated to 95 °C to stop all enzyme activity and then cooled down to room temperature. The cooled mixtures were filtered through two layers of cheese cloth and the filtrate was centrifuged at 10,000× *g* for 40 min at 4 °C. The filtrate was adjusted to pH 7.0 and then filtered (Whatman grade 3) on a Buchner funnel. The solutions were sterilized in an autoclave and spun down again.

The resulting clear mixture was used instead of tryptone and yeast extract in the glycerol BM (basal medium—see Test Strain and Chemicals), giving BYPP media with final protein concentrations of on average 2.18 g/L for lung and 2.48 g/L for spleen.

### 3.5. Protein Measurements

Protein was estimated by measuring the optical density of the clear solutions in a spectrophotometer (Epoch, BioTek Instruments Inc., Winooski, VT, USA) at 280 nm (OD_280_) [28]. To standardize the measurements, solutions of the digests (from both lung and spleen) were analyzed using the Kjeldahl method (*n* × 6.25) [29]. The linear regression gave the following equations (y = a + bx, where y = protein, g/L and × = OD_280_) (Equation (1) is for lung and (2) for spleen):

y = 0.0007 + 0.1715 × (R^2^ = 0.9973), (1)

y = 0.0007 + 0.1818 × (R^2^ = 0.9979), (2)

Protein was also estimated using Bradford’s method [24] using 96% clean bovine serum albumin (Sigma-Aldrich) and Bradford reagent [30]. The linear regression gave the following equation (Equation (3) (x = BSA, g/L and y = OD_595_).

y = −0.0195 + 0.3213 × (R^2^ = 0.9769), (3)

Theoretical maximum protein yield in the different mixtures was estimated with information from the United States Department of Agriculture, Food Data Central [31] (Table 1).

### 3.6. Growth Studies

Inoculation for growth studies was prepared by transferring a colony of *S minutum* from a BM agar plate to a 20 mL glucose BM, which was then incubated at 25 °C for 7 days at 150 rpm. Thereafter, all 20 mL volumes were transferred to 100 mL of glycerol BM and incubated at 25 °C for 3 days at 150 rpm. The three-day old cultures were combined and adjusted to 0.300 OD_660_ nm. Ten milliliters of this dilution was used to inoculate 90 mL of BM and 90 mL of media made from BYPP solutions. The flasks were incubated at 25 °C, with rotation at 150 rmp. After 5 days, the cultures were spun down, the cell pellet washed three times (two times with about 25 mL of 1% NaCl and once with about 5 mL of O) and then freeze dried. Growth was estimated as dry weight biomass (g/L).

### 3.7. Fatty Acid Analysis

For fatty acid analysis, *S. minutum* was grown in a separate experiment from the growth studies. *S. minutum* was grown, as described in the chapter on growth studies, in 6 flasks of glycerol BM and in 3 flasks containing BYPP lung media. BYPP solution from lung digested with pancreas was also analyzed to investigate whether the solution contained fatty acids, as this could influence the fatty acid composition of *S. minutum* after growth studies. Fatty acids measured in the BYPP solution from lungs digested with pancreas were mostly C16 or shorter and did not influence analyses of biomass (data not shown).

Lipid extractions were performed before methylations. Freeze-dried biomass (around 0.06 g) was crushed with a mortar and pestle and then placed in tubes with a Teflon screw cap, Tris-EDTA (pH 7 at 20 °C), chloroform, and methanol were added (1.6 mL, 2 mL and 4 mL, respectively), with 20 s vortex performed after addition of each chemical before the mixtures were incubated for an hour at room temperature. Two milliliters of chloroform and two milliliters of Tris-EDTA were added separately with 20 s vortex in between, before the glass tubes were centrifuged at 2000× *g* at 4 °C for 5–10 min. Bottom-layer lipids were carefully transferred with a Hamilton syringe to dry glass and N2 gas was used to evaporate the solvent before the lipid-containing glass containers were dried at 50 °C for 15 min and then cooled in desiccators. Cool dried glasses were weighed and the total lipid content was estimated.

Methylation of the lipids extracted to fatty acid methylesters (FAMEs) was carried out by adding a trans-esterification solution (containing 30 mL methanol, 3.0 mL chloroform, and 1.0 mL concentrated H_2_SO_4_) and vortexing briefly before the mixtures were incubated for 90 min at 90 °C. After cooling, FAMEs were extracted three times using 2 mL isooctane and the combined extracts were washed with 2 mL of H2O. Sodium sulfate was used to dry the solvent before glass containers were centrifuged for five minutes at 2000× *g* at 4 °C and FAMEs were transferred to GS-instrument vials. Gas Chromatography–Flame Ionization Detection was used for fatty acid composition analysis.

### 3.8. Statistical Analyses

Statistical analyses were performed using R [32] and SAS[33]. Data from growth studies were calculated in g/L and then compared. ANOVA was used to determine if there were any significant differences between treatments, and Tukey’s test was used to locate the differences. The linear ranges of protein concentrations and OD_280_ nm and the initial protein concentration and dry weight biomass were calculated via linear regression analysis[33].

## Figures and Tables

**Table 1:** Typical composition of by-products used as a raw material to produce BYPP. Information was retrieved from the United States Department of Agriculture Food Data Central [31].

Nutrient (g per 100 g)	Lung (Beef)	Spleen (Beef)	Pancreas (Lamb)
Protein	16.2	18.3	14.8
Carbohydrate	0	0	0
Total lipid	2.5	3.0	9.8
Saturated	0.86	1.00	4.44
Monounsaturated	0.64	0.78	3.54
Polyunsaturated	0.34	0.22	0.48

## Data Availability

Data is available from the corresponding author.
